# P-1510. *In Vitro* Activity of Ceftibuten-Avibactam and Comparator Agents Against Enterobacterales Collected Globally as Part of the ATLAS Surveillance Program, 2022 by Region

**DOI:** 10.1093/ofid/ofae631.1679

**Published:** 2025-01-29

**Authors:** Mark Estabrook, Julie Dickson, Gregory Stone, Katherine Perez, Daniel F Sahm

**Affiliations:** IHMA, Schaumburg, Illinois; IHMA, Schaumburg, Illinois; Pfizer, Inc., Groton, Connecticut; Pfizer, Inc., Groton, Connecticut; IHMA, Schaumburg, Illinois

## Abstract

**Background:**

Ceftibuten is an orally administered third-generation cephalosporin in clinical development in combination with an oral prodrug of avibactam, an inhibitor of class A β-lactamases, including ESBLs and KPC, class C (AmpC) β-lactamases, and some class D (OXA-48) β-lactamases. This study evaluated the *in vitro* activity of ceftibuten-avibactam and comparators against Enterobacterales isolates collected globally as part of the 2022 Antimicrobial Testing Leadership and Surveillance (ATLAS) program, by region.

**Figure:**
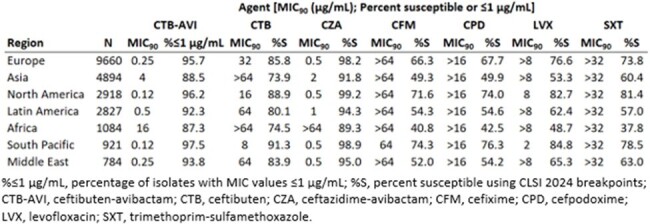

**Methods:**

23,088 non-duplicate clinical Enterobacterales isolates were collected in 2022 from 217 medical centers in 56 countries. Susceptibility testing was performed by CLSI broth microdilution and interpreted using CLSI 2024 breakpoints. Ceftibuten was tested with a fixed concentration of 4 µg/mL avibactam.

**Results:**

Ceftibuten-avibactam was active at ≤1 µg/mL against more isolates than were susceptible to any oral comparator agent (87.3-97.5%). Ceftibuten-avibactam also demonstrated the lowest MIC_90_ values, by region among the oral agents tested. Regional differences in the *in vitro* activity of ceftibuten-avibactam were observed with a lower MIC_90_ value against isolates collected in Europe, North America, the South Pacific, and the Middle East (0.12-0.25 µg/mL) as compared to Latin America, Asia, and Africa (0.5, 4, and 16 µg/mL, respectively). This phenomenon was also observed among oral comparator agents, with fewer isolates collected in Africa susceptible to cefixime (40.8%), cefpodoxime (42.5%), levofloxacin (48.7%), and trimethoprim-sulfamethoxazole (37.8%) than in any other region. A similar pattern was observed for the intravenously administered ceftazidime-avibactam, with 89.3% of isolates collected in Africa susceptible.

**Conclusion:**

Based on these data, ceftibuten-avibactam demonstrates promise as a candidate for oral treatment of infections caused by Enterobacterales. Regional trends in antimicrobial resistance to a diverse range of agents warrant continued monitoring.

**Disclosures:**

**Mark Estabrook, MS**, Pfizer, Inc.: Advisor/Consultant **Julie Dickson, BS**, Pfizer, Inc.: Advisor/Consultant **Daniel F. Sahm, PhD**, Pfizer, Inc.: Advisor/Consultant

